# Serum Autophagy-Related Protein 5 and Clinically Defined Cognitive Status in Older Adults: A Plate-Stratified Cross-Sectional Study Across: Normal Cognition, Mild Cognitive Impairment, and Alzheimer’s Disease

**DOI:** 10.3390/medicina62091722

**Published:** 2026-09-07

**Authors:** Kübra Erdoğan, Cemile Biçer, Rıdvan Erten, Kübra Kaya, Serap Boz, Hatice Turgut Şahin, Cemile Peker, Arzu Nevin Dağdemir, Büşragül Yılmaz, Aslıhan Yıldırım, Rana Tuna Doğrul, Hande Selvi Öztorun, Güneş Eken, Kamile Sılay

**Affiliations:** 1 Division of Geriatrics, Department of Internal Medicine, Ankara Bilkent City Hospital, 06200 Ankara, Türkiye; serapsen85@hotmail.com (S.B.); drhaticeturgut@gmail.com (H.T.Ş.); cemile.sanli.peker@gmail.com (C.P.); anevincoskun@gmail.com (A.N.D.); drbusragulyilmaz@gmail.com (B.Y.); g.asliyildirim@gmail.com (A.Y.); rana_tuna@hotmail.com (R.T.D.); 2 Department of Medical Biochemistry, Faculty of Medicine, Ankara Yıldırım Beyazıt University, 06760 Ankara, Türkiye; cbicer@aybu.edu.tr; 3 Division of Geriatrics, Department of Internal Medicine, Faculty of Medicine, Kocaeli University, 41380 Kocaeli, Türkiye; dr.ridvanerten@gmail.com; 4 Department of Translational Medicine, Faculty of Medicine, Ankara Yıldırım Beyazıt University, 06760 Ankara, Türkiye; kubrafidanb@gmail.com; 5 Division of Geriatrics, Department of Internal Medicine, Faculty of Medicine, Ankara Yıldırım Beyazıt University, 06760 Ankara, Türkiye; drhandeslv@hotmail.com (H.S.Ö.); kamilesilay@hotmail.com (K.S.); 6 Division of Geriatrics, Department of Internal Medicine, Faculty of Medicine, Health Science University, 06230 Ankara, Türkiye; guneseken@yahoo.com

**Keywords:** autophagy, ATG5, mild cognitive impairment, Alzheimer’s disease, Standardized Mini Mental State Examination, comprehensive geriatric assessment

## Abstract

*Background and Objectives*: Impaired autophagy has been implicated in neurodegeneration, but circulating autophagy-related proteins have shown inconsistent associations with cognitive impairment. We compared serum autophagy-related protein 5 (ATG5) among older adults with normal cognition, mild cognitive impairment (MCI), and clinically diagnosed Alzheimer’s disease (AD), and examined its relationship with global cognitive performance. *Materials and Methods*: This single-centre cross-sectional study enrolled 164 older adults (55 with normal cognition, 55 with MCI, and 54 with clinically diagnosed AD). Cognitive status was determined through integrated clinical assessment rather than a single test cut-off. An enzyme-linked immunosorbent assay was employed to quantify serum ATG5, yielding quantifiable values for 156 participants, right-censored observations above the highest calibrator for six, and unmeasurable results for two. Since the two 96-well plates, drawn from one ELISA kit lot and processed on the same day, exhibited a substantial discrepancy in measurement scale across runs, they were analysed as distinct strata, with the principal group contrast estimated via a right-censored Tobit regression of log-transformed ATG5 within each plate, controlling for cognitive group, age and sex; a pooled plate-adjusted model also provided support. Supportive analyses used within-plate z-standardised ATG5. Associations with clinical variables were assessed using age- and sex-adjusted partial Spearman correlations with false discovery rate correction. *Results*: Across both analytical strata (plate 1 omnibus *p* = 0.293; plate 2 *p* = 0.264) and within the supportive pooled model (*p* = 0.143; AD vs. normal geometric mean ratio 0.89, 95% CI 0.73–1.09), serum ATG5 concentrations did not differ significantly between cognitive groups. Concentrations on plate 2 were 47% lower (GMR 0.53, 95% CI 0.45–0.62), a gap exceeding the inter-assay imprecision declared by the manufacturer and unexplained by the calibrator values from plate 2; since relative dispersion matched across both plates (likelihood ratio *p* = 0.730), the pattern suggests a multiplicative scale shift of unknown origin. Serum ATG5 levels were weakly associated with global cognitive performance assessed via the S-MMSE (partial ρ = 0.283, *p* < 0.001, FDR *p* = 0.010), an association that persisted after adjusting for education, albumin and folate and retained a similar magnitude within a linear model (β = 0.044, *p* = 0.014). Incorporating ATG5 into a model that included age, sex and education failed to enhance discrimination (ΔAUC +0.012 and +0.000). *Conclusions*: Serum ATG5 did not discriminate normal cognition, MCI, and clinically diagnosed AD. A weak rank-based association with S-MMSE was observed, but the effect was small. These findings do not support serum ATG5 as a diagnostic discriminator for clinically defined cognitive status.

## 1. Introduction

Cognitive impairment and dementia are among the most consequential conditions of later life, with substantial effects on affected individuals, caregivers, and health systems [1]. Clinical categories such as mild cognitive impairment (MCI) and dementia aggregate biologically heterogeneous processes, and a single label may correspond to several underlying pathologies [2]. Because definitive pathological confirmation is often unavailable in routine geriatric care, peripheral biomarkers reflecting pathways linked to aging, neurodegeneration, inflammation, and cellular homeostasis remain of interest.

Macroautophagy (hereafter autophagy) is an evolutionarily conserved intracellular degradation pathway central to proteostasis, mitochondrial quality control, and clearance of aggregation-prone proteins and damaged organelles [3,4]. Autophagic activity declines with age, and its impairment has been mechanistically linked to accumulation of amyloid-β and tau and to neurodegeneration [3,4]. Autophagy-related protein 5 (ATG5) is an essential mediator of autophagosome formation and has been examined as a candidate peripheral marker. It should be emphasised that serum ATG5 is a peripheral measurement that may not directly reflect brain autophagic flux; mechanistic inferences from circulating levels are therefore limited.

Previous studies of circulating autophagy-related proteins in cognitive impairment are heterogeneous and, in places, contradictory. Reduced serum ATG5 and mitophagy markers have been reported in Alzheimer’s disease (AD) and MCI relative to controls [5], whereas increased plasma ATG5 has been reported in the same conditions [6]; in cerebrovascular disease, higher serum ATG5 has been associated with poorer and declining cognition [7]. This inconsistency spans sample matrices, assay platforms, diagnostic definitions, and disease subtypes, and raises the possibility that assay-level factors contribute to divergent findings. Evidence is particularly limited to geriatric cohorts in which cognitive markers are interpreted together with functional, nutritional, depressive, renal, inflammatory, and metabolic variables.

We hypothesized that peripheral ATG5 might reflect differences in cognitive performance among older adults but might not necessarily discriminate clinically defined diagnostic categories, given the biological heterogeneity of geriatric cognitive impairment. Consequently, this study compared serum ATG5 among older adults with normal cognition, MCI, or clinically diagnosed AD using plate-adjusted and censoring-aware methods, and examined the association of serum ATG5 with global cognitive performance within a comprehensive geriatric assessment context.

## 2. Materials and Methods

### 2.1. Study Design and Participants

This was a single-centre, cross-sectional observational study of older adults evaluated in a geriatric clinic, comprising three groups: normal cognition, MCI, and AD. Cognitive status was established through integrated clinical assessment including medical history, caregiver information when available, cognitive testing, functional evaluation, and neuroimaging findings rather than by a cognitive test cut-off alone. Enrolment employed stratified consecutive sampling, whereby eligible attendees within each diagnostic stratum were recruited sequentially towards a pre-specified quota of approximately 55 per group; the final analytic cohort comprised 164 unique participants (normal cognition 55, MCI 55, and clinically diagnosed AD 54). Reporting followed the STROBE statement for cross-sectional studies [8].

### 2.2. Diagnostic Criteria

Normal cognition was defined as the absence of clinical evidence of cognitive impairment after geriatric and cognitive assessment. MCI was diagnosed according to Petersen’s criteria [9], requiring a cognitive concern (supported by patient or informant history), objective cognitive impairment relative to age and education where available, largely preserved basic activities of daily living, and absence of dementia. AD was diagnosed after comprehensive geriatric and cognitive evaluation according to the NINCDS ADRDA [10] and DSM-5 [11] criteria. Brain magnetic resonance imaging (MRI) was reviewed or performed when clinically indicated as part of the diagnostic work-up to exclude alternative causes of cognitive impairment. MRI supported exclusion of alternative causes but did not provide molecular confirmation of AD pathology; the AD group therefore denotes clinically diagnosed AD and should not be interpreted as biomarker-confirmed AD.

### 2.3. Exclusion Criteria

Individuals were excluded for active inflammatory conditions, acute infection, active malignancy, advanced or decompensated heart failure, vascular dementia, Parkinson’s disease dementia, dementia with Lewy bodies, frontotemporal dementia, or other neurodegenerative disorders, and for any condition that could substantially interfere with cognitive evaluation or the interpretation of serum ATG5. A history of stroke was not itself an exclusion criterion unless the clinical and neuroimaging evaluation supported vascular dementia as the primary diagnosis. Because non-AD dementias were excluded, the diseased group represents clinically diagnosed AD rather than all-cause dementia, which should be reflected when generalizing the findings.

### 2.4. Comprehensive Geriatric and Cognitive Assessment

As part of the comprehensive geriatric assessment, basic and instrumental activities of daily living were evaluated with the Katz Activities of Daily Living scale (ADL) [12] and the Lawton Brody Instrumental Activities of Daily Living scale (IADL) [13]; depressive symptoms with the short form of the Yesavage Geriatric Depression Scale (GDS-15) [14]; malnutrition risk with the Mini Nutritional Assessment–Short Form (MNA-SF) [15]; and global cognitive performance with the Standardized Mini Mental State Examination (S-MMSE) [16]. Cognitive classification integrated clinical history, caregiver information, S-MMSE performance, functional status, and diagnostic criteria rather than relying on the S-MMSE score alone. Nutritional and depressive symptom scales were treated as clinical correlates instead of diagnostic criteria, while functional measures and the S-MMSE may aid in the clinical characterization of cognitive groups. Among the 17 participants in whom the S-MMSE could not be completed (14 with AD, 1 with MCI, and 2 with normal cognition), the severity of cognitive impairment precluded reliable testing or communication. These observations reflect non-assessability rather than missingness at random; consequently, they were excluded from the S-MMSE-based analyses instead of being imputed, and no floor value was assigned.

### 2.5. Collected Clinical Variables

Recorded variables included age, sex, living arrangement, educational status, smoking status, alcohol use, body mass index, comorbidities, urinary incontinence, history of falls, and number of medications. Only variables with adequate data completeness were analysed; those with substantial missingness are noted below. Educational status was recorded as an ordinal level (from no formal schooling to university) and was available for nearly all participants; because it was available, education-adjusted sensitivity analyses were performed for the ATG5 S-MMSE association and for the group comparison.

### 2.6. Serum ATG5 Measurement

Serum ATG5 was measured by ELISA using a commercially available kit (SunLong Biotech Co., LTD, SL2770Hu, Hangzhou, China). Assays were carried out on two 96-well plates derived from a single kit lot (SunLong Biotech, Cat. SL2770Hu, Lot 20250704), performed in the same laboratory by the same operator under the same protocol, yet the two plates remained separate assay runs, with calibrators assayed in duplicate on plate 1 but in singlicate on plate 2. The kit used a sandwich ELISA with an on-plate detection range of 0.1–10 ng/mL and an analytical sensitivity of 0.01 ng/mL, with intra- and inter-assay coefficients of variation (CV) below 10% and 12%, respectively, in accordance with manufacturer specifications; serum was assayed at the recommended 1:5 dilution, and the calibrators actually run (9, 6, 3, 1.5 and 0.75 ng/mL on plate) gave a calibrated serum range of 3.75–45 ng/mL (further assay and plate details are provided in Supplementary Table S1). Although both plates originated from a single kit lot and employed calibrators from that same lot, ensuring that any discrepancy in measurement scale stems from run-to-run (between-plate) variation instead of differences between reagent lots, the observed between-plate difference nonetheless surpassed the manufacturer’s specified upper limit for inter-assay imprecision. Because a marked disparity in measurement scale was detected between the two plates, raw serum ATG5 concentrations from different plates were not combined without applying statistical adjustment. Six samples that surpassed the maximum standard curve calibrator (9 ng/mL on plate; 45 ng/mL for serum after the 1:5 dilution) were right-censored during the primary analysis. Samples flagged during quality control as lipaemic or clotted were retained but examined in a sensitivity analysis. Routine biochemistry and haematology were obtained as part of clinical care.

### 2.7. Statistical Analysis

Median and interquartile range (IQR) are reported for continuous variables, whereas counts and percentages are used for categorical variables. The Kruskal–Wallis test was applied to continuous variables, while the chi-square or Fisher’s exact test was used for categorical variables in the three-group comparisons. The Benjamini–Hochberg FDR procedure was applied to control multiplicity within test families [17]. Given the substantial difference in measurement scale between the two ELISA plates, the principal group comparison employed a right-censored (Tobit) regression on log-transformed serum ATG5, estimated independently for each plate with cognitive group, age and sex included as covariates and normal cognition designated as the reference; a combined model incorporating the ELISA plate was also fitted to provide supportive evidence. A Wald omnibus test was used to evaluate the group effect, while serum values exceeding the top standard curve calibrator (45 ng/mL) were treated as right-censored [18]. Plate allocation was balanced across cognitive groups by design, with near-equal numbers from each group on each plate, so plate was not confounded with cognitive group. Within a supportive analysis, the Kruskal–Wallis test was used to compare within-plate z-standardised ATG5. Associations between ATG5 and continuous clinical/laboratory variables used partial Spearman correlations adjusted for age and sex, with an additional sensitivity model adjusted for folate; because educational status was available, the ATG5 S-MMSE association was also adjusted for education, alone and together with folate. As a supplementary model, linear regression was fitted, and a cubic-spline likelihood-ratio test was used to examine departure from linearity. Secondary analyses employed binary logistic regression to assess whether within-plate z-standardised ATG5 showed an independent association with AD (versus normal plus MCI) and cognitive impairment (MCI plus AD versus normal), with models adjusted for age, sex, and educational level, and a further model also incorporating folate; cognitive and functional scores used in the diagnostic process were deliberately omitted to prevent circularity. The area under the receiver operating characteristic curve (AUC), accompanied by bootstrap 95% confidence intervals, was used to summarise discriminative performance; a diagnostic cut-off was not derived since absolute ATG5 values could not be compared between the two analytical runs, which produced systematically different concentrations despite both plates originating from a single kit lot. Possible nonlinearity of the ATG5 S-MMSE relationship was tested by comparing a linear model with a cubic-spline model via a likelihood ratio test. Quality-control-flagged samples were omitted in the sensitivity analyses, each plate was evaluated independently, a plate-by-group interaction was examined, and the key associations were further adjusted for educational level. Analyses used Python 3 (SciPy 1.16. 3 and statsmodels 0.14.6).

### 2.8. Missing Data

Missingness was variable specific and analyses used available cases; no imputation was performed. Erythrocyte sedimentation rate and structured medication category fields had high missingness and were excluded. For the plate 1 subgroup, medication data were reconstructed from unstructured records and were therefore considered insufficiently standardised for inferential analysis and were not analysed further.

## 3. Results

### 3.1. Participant Characteristics

**Figure 1:**
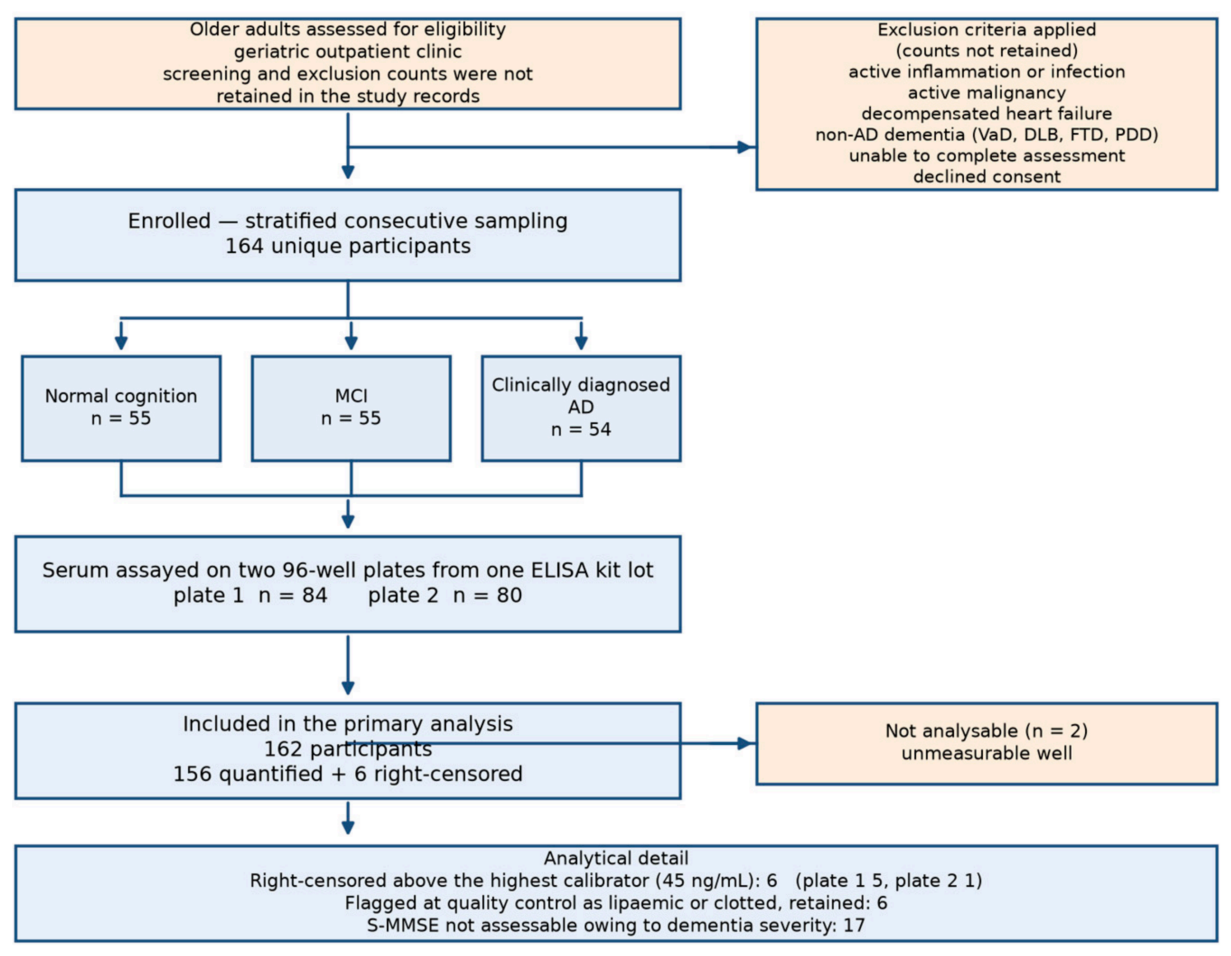
Participant flow diagram (STROBE). Stratified consecutive sampling was used to enrol 164 unique older adults from those screened in the geriatric clinic, comprising 55 with normal cognition, 55 with MCI, and 54 with clinically diagnosed AD. ATG5 concentrations in serum were determined using two 96-well plates derived from a single kit lot belonging to one ELISA catalogue (SunLong Biotech, Cat. SL2770Hu). The assays were performed consecutively by a single operator in one laboratory during a single working session (plate 1, n = 84; plate 2, n = 80); thus, these two plates constitute two analytical runs of a single reagent lot rather than two distinct reagent lots. After two samples proved unmeasurable, 162 participants remained with either a quantifiable (n = 156) or right-censored (n = 6) value. Six observations exceeded the highest calibrator (45 ng/mL) and were treated as right-censored. Six samples flagged at quality control as lipaemic or clotted were retained and examined in a sensitivity analysis. The number of individuals screened and the number excluded by reason were not retained in the study records; the exclusion criteria applied are listed in the figure.

Of the 164 unique older adults in the analytic sample (normal cognition 55, MCI 55, clinically diagnosed AD 54; Figure 1), 156 had serum ATG5 quantified, six had values right-censored because they exceeded the highest calibrator (45 ng/mL), and two had unmeasurable levels, resulting in 162 participants with a quantifiable or censored value. For one participant, whose blood was drawn on two occasions a year apart, only the initial measurement was retained, ensuring that this individual contributes a single value to the analysis. In contrast to the normal cognition (78.0) and MCI (78.0) groups, the AD cohort had a higher median age of 82.0 years (*p* = 0.008), whereas sex distribution was similar (female: 64%, 71%, 63%; *p* = 0.623). S-MMSE and functional/nutritional scores differed markedly across groups (Table 1); because these instruments partly inform group definitions, these differences are reported as group characterization.

### 3.2. Laboratory and Clinical Profile

Among laboratory variables, the AD group showed lower total protein, albumin, and alanine aminotransferase (ALT) (FDR significant; Table 1), compatible with a poorer nutritional or systemic-health profile but not establishing sarcopenia, which was not directly assessed. Renal function, glucose, HbA1c, and bone densitometry T scores did not differ across groups, and no comorbidity differed after FDR correction.

### 3.3. Primary ATG5 Analysis

**Figure 2:**
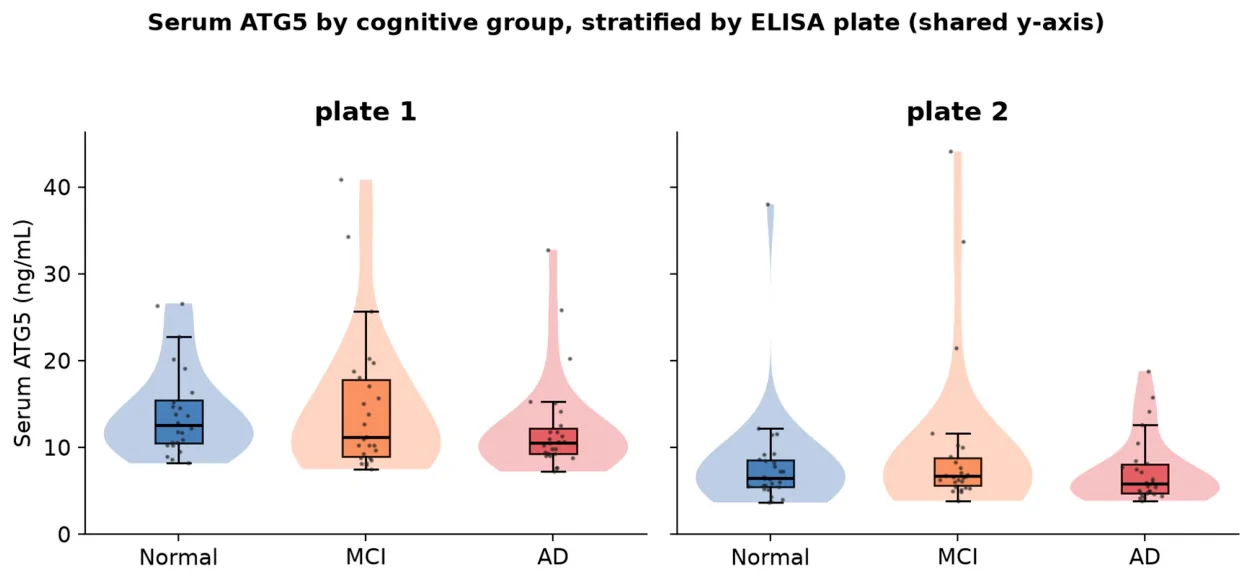
Serum ATG5 (ng/mL) levels across cognitive groups, stratified by ELISA plate, are shown with a shared y axis. The evident between-plate scale difference indicates the requirement for plate-adjusted analysis, whereas distributions overlapped substantially across the normal cognition, MCI, and AD groups within each plate. Boxes show median and IQR; violins show the distribution; points are individual observations. Six values above the highest calibrator (45 ng/mL) are not displayed.

Within both analytical strata, serum ATG5 concentrations showed no significant differences between cognitive groups (plate 1: omnibus group *p* = 0.293; geometric mean ratio [GMR] for MCI relative to normal 0.96, 95% CI 0.72–1.26; AD versus normal GMR 0.81, 95% CI 0.62–1.07; plate 2: omnibus *p* = 0.264; MCI GMR 1.19, 95% CI 0.90–1.58; AD GMR 0.95, 95% CI 0.71–1.28). The pooled plate-adjusted model, which provided supportive evidence, yielded concordant results (omnibus *p* = 0.143; MCI GMR 1.09, 95% CI 0.90–1.33; AD GMR 0.89, 95% CI 0.73–1.09), and no plate-by-group interaction was detected (*p* = 0.589; Table 2). The concentration values recorded on plate 2 fell systematically below those on plate 1 (GMR 0.53, 95% CI 0.45–0.62). We re-estimated the Tobit model by introducing a plate-specific residual variance to investigate this disparity, finding that the dispersion parameters were nearly identical (σ = 0.507 and 0.527 on the log scale; likelihood ratio χ^2^(1) = 0.12, *p* = 0.730), suggesting the divergence between plates arose from a multiplicative shift in scale rather than from variability in the measured signals. As the two plates were sourced from one kit lot and quantified using that lot’s calibrators, the discrepancy signifies a run-to-run (between-plate) shift in the measurement scale, and the standard curve does not explain it and would, if anything, act in the opposite direction. Because the plate 2 calibrators produced optical densities on average about 25% lower than those on plate 1 at the same nominal concentrations, the plate 2 curve assigns a higher concentration to any given optical density. Interpolating the plate 1 median signal on the fitted plate 2 curve gives 3.14 ng/mL on the plate rather than 2.20 ng/mL; because the two curves differ in slope, this shift is not a constant multiple across the calibration range. The measured concentrations differed 1.73-fold in the opposite direction, which places the difference in the sample signal itself rather than in the calibration. A discrepancy of this magnitude between plates corresponds to approximately four times the upper limit for inter-assay imprecision specified by the manufacturer (coefficient of variation below 12%), and while its cause could not be definitively determined in a retrospective review, the two runs exhibited one documented variation: plate 1 calibrators were read in duplicate whereas those on plate 2 were read in singlicate, meaning the plate 2 calibration curve was based on single wells and was consequently less well constrained. Since every sample underwent singlicate analysis on both plates without any duplicate measurement, it was impossible to calculate in-house intra-assay or inter-assay coefficients of variation, meaning the values cited here represent manufacturer specifications instead of data generated in our laboratory. A supportive comparison of within-plate z-standardised values was concordant with the primary analysis (Kruskal–Wallis *p* = 0.095; Figure 2). There was thus no evidence that serum ATG5 discriminates among these cognitive categories.

### 3.4. Association Between Serum ATG5 and S-MMSE

**Figure 3:**
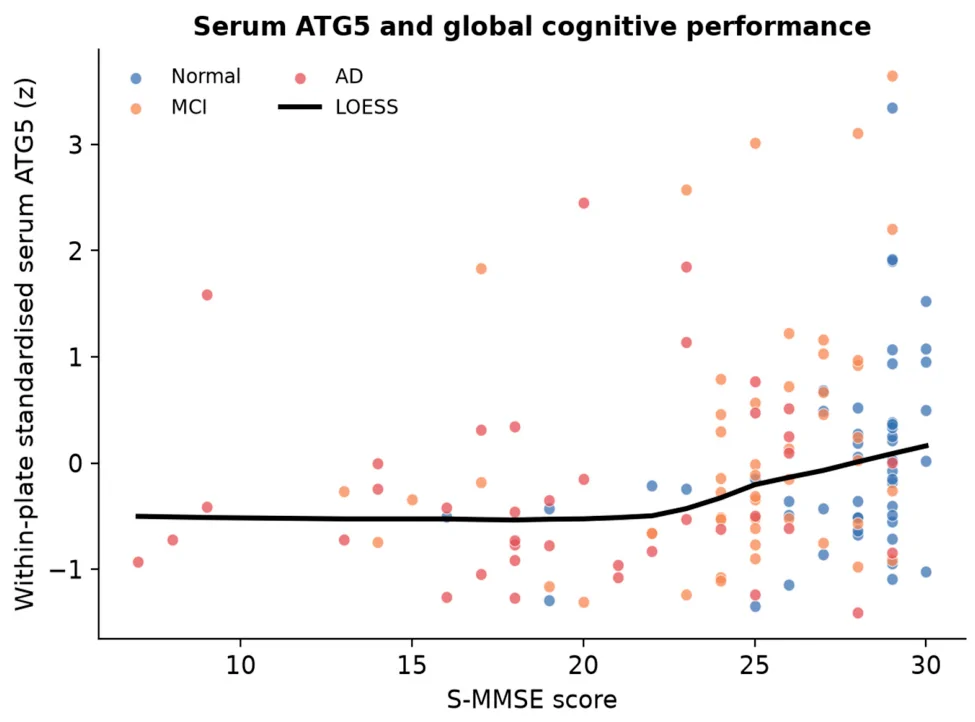
Within-plate standardised serum ATG5 versus S-MMSE score with a LOESS smoother. Both linear and rank-based approaches indicated a weak but consistent association (partial ρ = 0.283; β = 0.044, *p* = 0.014), and there was no evidence of departure from linearity (spline likelihood-ratio *p* = 0.402). Applying standardisation eliminates the scale discrepancy between plates.

Although no group-level disparity was detected, elevated serum ATG5 demonstrated a weak association with superior global cognitive performance. The partial Spearman correlation, adjusted for age and sex, revealed an association between ATG5 and S-MMSE (ρ = 0.283, 95% CI 0.122–0.429, *p* < 0.001, FDR *p* = 0.010; n = 139; Table 3). Adjusting for education (ρ = 0.295), education alongside albumin (ρ = 0.259), or education and albumin plus folate (ρ = 0.292; all *p* ≤ 0.002; Table 4) retained the association, which showed a comparable effect size in a linear model (β = 0.044, 95% CI 0.009–0.079, *p* = 0.014). The cubic-spline model did not improve on the linear fit (likelihood-ratio *p* = 0.402), providing no indication of deviation from a monotonic, approximately linear association of modest effect size (shared variance ≈ 8%; Figure 3). Among cognitive and functional variables, only S-MMSE remained significant after FDR correction across the full panel; the Lawton IADL, albumin, and corrected calcium signals were nominal only and are reported as exploratory. Serum ATG5 showed no significant association with estimated GFR, C-reactive protein, or vitamin B12. ATG5 also correlated with folate (ρ = 0.255, FDR *p* = 0.015), an association that is discussed below.

### 3.5. Secondary and Sensitivity Analyses

**Figure 4:**
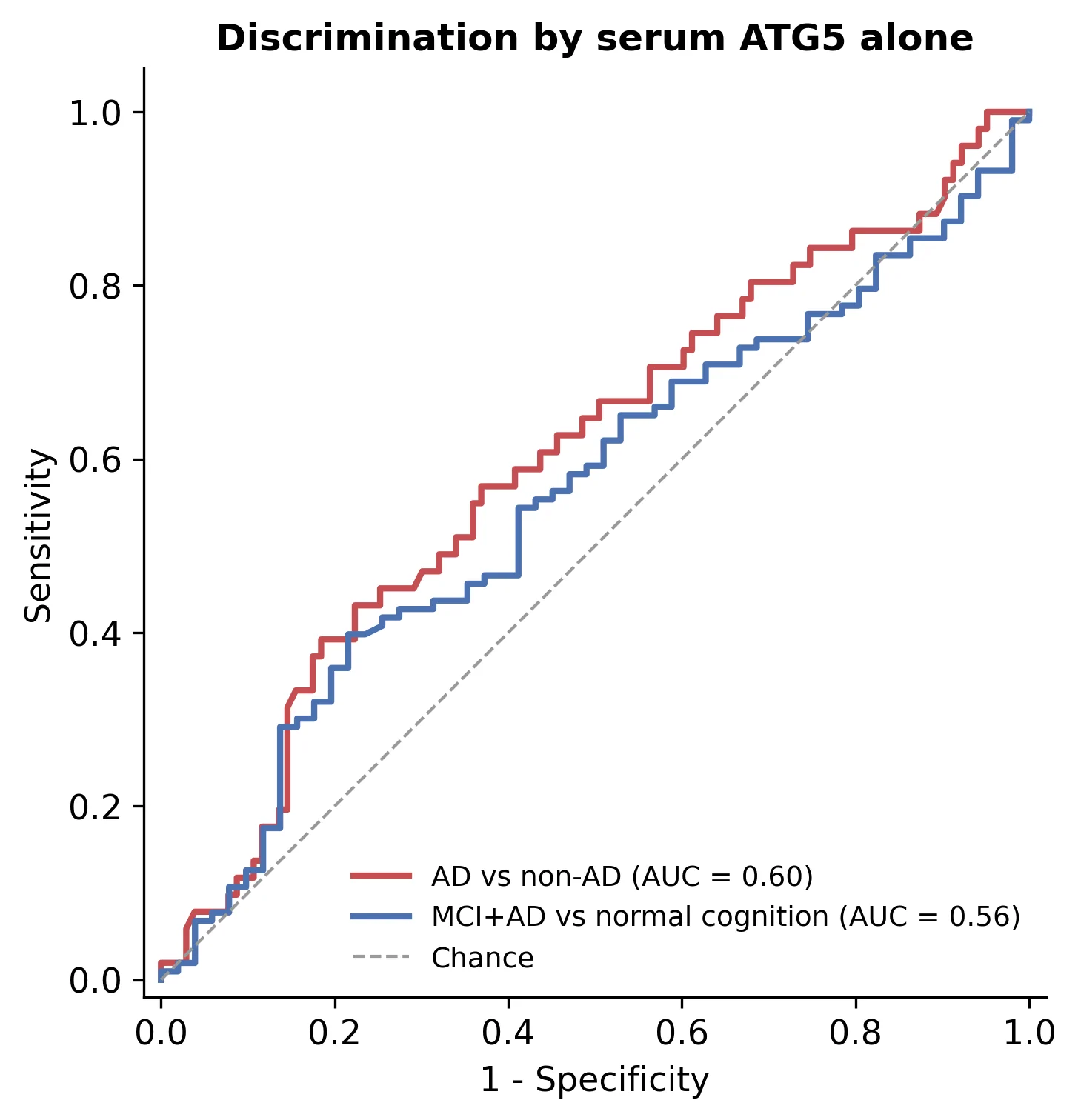
Receiver operating characteristic curves for within-plate standardised serum ATG5. Discrimination was poor for both contrasts (clinically diagnosed AD vs. normal cognition or MCI, AUC = 0.60; MCI + AD versus normal cognition, AUC = 0.56). No clinical cut-off is proposed because absolute ATG5 values were not comparable across the two plates and discriminatory performance was poor.

After adjusting for age, sex, and educational level in binary logistic regression, within-plate z-standardised ATG5 showed no independent association with clinically diagnosed AD versus normal cognition or MCI (odds ratio [OR] 0.75, 95% CI 0.50–1.12, *p* = 0.157), nor with cognitive impairment (MCI plus AD) versus normal cognition (OR 0.97, 95% CI 0.69–1.36, *p* = 0.854), a pattern that persisted following further folate adjustment. Discrimination was not improved when ATG5 was added to the base model including age, sex and educational level (area under the curve 0.700 to 0.712, ΔAUC +0.012, likelihood-ratio *p* = 0.141; and 0.601 to 0.601, ΔAUC +0.000, *p* = 0.854; Table 5). As exploratory evaluations of within-sample discrimination, these receiver-operating-characteristic analyses should be interpreted cautiously because recruitment from predefined diagnostic strata in a specialist clinic may yield a case spectrum that differs from routine clinical practice. The resulting estimates may therefore not represent expected performance in an unselected clinical population. Older age and lower educational level were associated with AD, as expected. The z-standardised ATG5 showed poor discriminatory ability (AUC 0.60, 95% CI 0.51–0.70 for clinically diagnosed AD versus others; AUC 0.56, 95% CI 0.47–0.65 for impairment versus normal cognition; Figure 4), precluding the derivation of a diagnostic cut-off because absolute ATG5 values were not comparable between the two plates and discriminatory performance was poor. The likelihood-ratio *p* = 0.402 indicated that a cubic-spline model provided no significant improvement over a linear fit for the ATG5 S-MMSE relationship, leading us to describe the association as weak yet consistent across linear and rank-based models.

Educational level was lower in the AD group (77% had at most primary education versus 60% in the normal and 53% in the MCI group; Kruskal–Wallis on ordinal level *p* = 0.023). The ATG5 S-MMSE association persisted after adjustment for age, sex, and education (partial ρ = 0.226, *p* = 0.005) and after additional folate adjustment (ρ = 0.225, *p* = 0.007). Sensitivity analyses confirmed the robustness of the primary null group comparison, as concordant results were obtained when quality-control flagged samples were excluded (Kruskal–Wallis *p* = 0.107), each plate was analysed separately (plate 1 *p* = 0.199; plate 2 *p* = 0.475), or education was adjusted for (group omnibus *p* = 0.36), with no plate-by-group interaction detected (*p* = 0.589).

## 4. Discussion

This study has two principal findings. First, in plate-stratified right-censored analyses, no significant variation in serum ATG5 was detected among older adults with normal cognition, MCI, and clinically diagnosed AD. Second, serum ATG5 showed a weak rank-based association with global cognitive performance measured by S-MMSE. Together, these results argue against serum ATG5 as a discriminator of cognitive diagnostic categories while leaving open a modest link with continuous cognitive performance. In secondary analyses, ATG5 provided no independent diagnostic contribution in logistic models and no clinically useful discriminative performance in ROC analyses, reinforcing the negative categorical finding.

Even with exclusion of vascular dementia, dementia with Lewy bodies, frontotemporal dementia, and Parkinson’s disease dementia, the clinical diagnosis of AD in this study was not confirmed by molecular biomarkers. Without amyloid/tau, cerebrospinal fluid, positron emission tomography, or plasma phosphorylated-tau confirmation, the AD group cannot be described as biomarker-confirmed. MRI was used to exclude alternative causes but does not establish AD pathology. Consequently, a peripheral marker reflecting a general homeostatic pathway may be diluted across residual pathological heterogeneity, attenuating group-level contrasts; the older age of the AD group, although adjusted for, may also leave residual confounding.

Our findings should be read against a discordant evidence base. Reduced serum ATG5 and mitophagy markers have been reported in AD and MCI [5], whereas increased plasma ATG5 has been reported in the same conditions [6], and higher serum ATG5 has been associated with poorer longitudinal cognition in cerebrovascular disease [7]. This inconsistency aligns with the acknowledged effects of sample type, assay platform, kit and lot, diagnostic criteria, patient selection, and pre-analytical handling on circulating autophagy-related measurements, factors usually managed analytically as batch effects [19]; nevertheless, the marked scale discrepancy detected in this study occurred across two plates from a single kit lot, processed by one operator within the same laboratory under nominally identical conditions using calibrators from that identical lot, which argues against kit or lot variation and leaves an unexplained between-plate, run-to-run variance that greatly exceeds the manufacturer’s specified maximum inter-assay imprecision (coefficient of variation below 12%). We avoided selective citation and note explicitly that the direction of association across studies is not consistent.

Another clinical study reported that serum ATG5, quantified via ELISA in 50 patients with Alzheimer’s disease and 50 controls, separated the cases from the controls with excellent receiver operating characteristic performance [20]; however, our data, derived from a larger and older sample evaluated across three cognitive strata, fail to replicate that finding. The substantial divergence between studies employing nominally the same analyte is hard to attribute to biological factors alone, pointing instead to assay-level and case-selection factors.

The data presented here illustrate the extent to which the assay itself may account for the observed between-study discordance. Although the two plates came from one and the same kit lot, and therefore from the same calibrator lot, and were processed by a single operator in one laboratory during a single assay session on a consecutively sampled population from a single collection period, they yielded systematically distinct concentrations, with median quantifiable values of 11.0 ng/mL (IQR 9.3–15.2) on plate 1 versus 6.3 ng/mL (IQR 5.2–8.5) on plate 2, and a geometric mean ratio of 0.53 (95% CI 0.45–0.62) for plate 2 relative to plate 1 in the pooled model adjusted for cognitive group, age and sex, representing a between-plate offset that substantially surpassed the manufacturer’s specified upper limit for inter-assay imprecision (coefficient of variation below 12%). That five of the six right-censored observations were found on plate 1 aligns with a shift of the entire distribution, as opposed to the influence of a few atypical individuals. The approximately two-fold difference associated with the analytical run, despite a common kit lot, calibrator lot, matrix, dilution, operator and population, exceeds several of the group differences that this literature has interpreted biologically. The existing literature draws on heterogeneous kits, lots, assay runs, matrices and dilution factors, with lot numbers, plate assignment and run dates rarely documented; given that the discrepancy detected here emerged between two plates from a single lot, specifying the lot in isolation cannot guarantee cross-study comparability of absolute circulating ATG5 levels, meaning the conflicting results for serum [5] and plasma [6] need not indicate divergent biology. The magnitude of this cross-study inconsistency is evident from the reported figures, as one cohort [5] provided median serum ATG5 of 45.95 ng/mL for controls and 12.35 ng/mL for Alzheimer’s disease, whereas a separate group [6] documented mean plasma ATG5 of 129.0 ng/mL for controls and 149.3 ng/mL for dementia, in contrast to the 11.0 and 6.3 ng/mL median serum results obtained on the two plates analysed here. The 1.73-fold difference observed between our plates is contextualised by the roughly twenty-fold variation in reported values for this nominal analyte, a discrepancy that endures even within a single matrix because the published serum control value is four to seven times higher than our own serum medians. Our data do not establish that assay variation is the cause of those findings, but rather that such variation is capable of producing discordance at the scale observed, and that any reading of absolute ATG5 concentrations remains uncertain until both run-to-run behaviour, the sole aspect assessable in the present study, and lot-to-lot behaviour have been fully characterised. The plate-stratified model serves as the principal analysis in this work, distinct from a conservative option, and we advise that subsequent investigations into circulating ATG5 document catalogue and lot numbers along with the specific plate allocation for each specimen, assay samples and calibrators in duplicate, retain the same replicate design on all plates rather than diminishing it during later sessions, add a shared control sample to every plate, and account for plate effects through stratification or adjustment; because the origin of the discrepancy noted here remained unidentified, these procedures aim to identify and measure such an artefact rather than to eliminate it.

Cautious interpretation is required for the association between serum ATG5 and S-MMSE. The presence of a detectable correlation alongside a null group comparison may be explained by the fact that a continuous cognitive score can retain more information than a categorical diagnosis. The association displayed similar magnitude within the normal-cognition and MCI strata and following group-demeaning, pointing to a within-group relationship instead of an artefact of between-group structure, yet it was null within the AD stratum, where exclusion of the most severely impaired participants restricts the S-MMSE range and limits the power of that stratum-specific estimate. Nonetheless, the effect was small and should not be considered clinically actionable. Educational level differed across groups and strongly influences S-MMSE performance; importantly, the ATG5 S-MMSE association persisted after adjustment for education, reducing the likelihood that it is explained by educational confounding. The association was essentially unchanged after additional adjustment for folate (ρ = 0.226 to 0.225), arguing against substantial confounding by folate; the correlation between ATG5 and folate nevertheless raises the possibility of a biological link involving one-carbon metabolism pathways; folate has been associated with cognitive performance in older adults, including with MMSE scores [21], and low folate with elevated homocysteine has been linked to MCI and AD risk [22]. These interpretations remain speculative and exploratory.

Interpreting a peripheral measurement benefits from the broader neurobiological context offered by ATG5. In the central nervous system, ATG5 has been most directly examined in microglia, where autophagy limits inflammatory signalling. In mice, myeloid lineage-specific Atg5 deletion generates a Parkinson disease-like phenotype characterised by defective motor coordination and cognitive learning, depletion of tyrosine hydroxylase-positive neurons and heightened neuroinflammation, a mechanism that involves accelerated NLRP3 inflammasome activation and interleukin-1β release and is reversed by the NLRP3 inhibitor MCC950 [23]. In 5 × FAD mice, the microglial ablation of ATG5 within an Alzheimer disease model was associated with diminished postnatal hippocampal neurogenesis and hastened neurodegeneration [24]. Two caveats warrant consideration. First, the available data pertain to intracellular ATG5 in a specific cell type, unlike our serum assessment of an immunoreactive signal with an unknown cellular source; also, the circulating steady-state level of one ATG protein does not quantify autophagic flux. Second, the reported direction of effect for microglial ATG5 varies across models, reflecting the inconsistency observed among circulating ATG5 studies.

The principal advantages comprise a balanced three-group design, a geriatric assessment extending beyond cognition, the exclusion of major non-AD dementias alongside inflammatory and malignant conditions, the application of MRI to rule out alternative causes within the diseased group, and a transparent, plate-adjusted and censoring-aware analytic approach, FDR control, and sensitivity analyses. These features support the internal validity of the negative primary findings.

Rather than being characterised as a null result, the precision of this study ought to be articulated explicitly. A residual standard deviation of 0.52 for log-transformed ATG5 translates to a geometric coefficient of variation of approximately 55%. Given the average of 54 participants per group, the design possessed 80% power to identify a shift in geometric mean concentration of roughly +32% or −24%, resulting in confidence intervals of commensurate width. Consequently, the observed results permit a range from a 27% reduction to a 9% increase for clinically diagnosed AD, and an increase of up to 33% for MCI. The plasma differences of 16% to 19% reported previously [6] were of a size this sample was underpowered to detect. Consequently, although the present results rule out a large disparity between groups, they do not demonstrate equivalence. Since ATG5 serves as the dependent variable, nondifferential random measurement error in the outcome would be expected to reduce precision rather than to bias the estimated group contrasts systematically.

Limitations must be emphasised. The cross-sectional design precludes causal or temporal inference. The study was single-centre with a modest sample size, and the ATG5 S-MMSE effect was weak. No amyloid/tau, cerebrospinal fluid, positron emission tomography, plasma phosphorylated-tau, neurofilament light, or glial fibrillary acidic protein measurements were available; the AD diagnosis was clinical only. Educational level, a strong determinant of S-MMSE, was available and included in adjusted analyses, and the ATG5 S-MMSE association was robust to it; nevertheless, unmeasured confounding cannot be excluded. Serum ATG5 was assayed across two ELISA plates from one kit lot, yet the resulting runs showed a systematic scale divergence exceeding the manufacturer’s stated inter-assay imprecision, an offset for which the analytical cause remained unidentified with certainty in retrospect. This offset is unlikely to generate or conceal a group difference. Because participants from each cognitive group were distributed across the two plates in near-equal numbers (28 with AD, 28 with MCI and 26 with normal cognition on plate 1; 26, 27 and 27 on plate 2), any purely multiplicative shift is cancelled within-plate, consistent with the primary model being stratified by plate, and no plate-by-group interaction was detected (*p* = 0.589). The offset does, however, limit confidence in absolute ATG5 values, and none of the arguments presented in this paper depend on such values. Pre-analytical variability is possible, and serum ATG5 may not reflect brain autophagic flux. Finally, exclusion of non-AD dementia subtypes limits generalizability to all-cause dementia. In our laboratory, the analytical specificity of the ELISA employed in this study has not undergone independent verification. Although the assay captures an immunoreactive signal assigned to ATG5, the biological identity of the measured analyte remains unverified in the absence of orthogonal confirmation, such as immunoblotting, mass spectrometry, spike recovery or dilutional linearity. Interpretation of both positive and negative findings should be moderated, as this limitation may also apply to published serum ATG5 measurements employing comparable kits.

Finally, the present investigation is wholly observational and provides no mechanistic insight. Autophagic flux, autophagosome turnover and any cell-type-resolved readout were not measured, and no cerebrospinal fluid data were available. As the cellular source of circulating ATG5 remains unknown, the cross-sectional design is unable to establish temporality or the direction of effect.

In terms of implications, serum ATG5 is not supported as a stand-alone diagnostic discriminator of cognitive categories. Should the weak S-MMSE association prove genuine, it warrants additional inquiry employing assays with independently validated inter-assay performance rather than reliance on manufacturer specifications, duplicate sample runs incorporating shared bridging controls on each plate, longitudinal cognitive assessments, and AD phenotypes defined by biomarkers.

## 5. Conclusions

In this cross-sectional study, serum ATG5 did not significantly differ among older adults with normal cognition, MCI, and clinically diagnosed AD. Higher serum ATG5 showed a weak association with S-MMSE performance that was consistent across rank-based and supplementary linear analyses; the effect was nevertheless small. These findings do not support serum ATG5 as a stand-alone diagnostic discriminator, although they suggest a limited, exploratory association between serum ATG5 immunoreactivity and global cognitive performance. Longitudinal studies using standardised ATG5 assays and biomarker-characterised AD phenotypes are needed.

## Figures and Tables

**Table 1:** Baseline characteristics by cognitive group.

Characteristic	Normal (n = 55)	MCI (n = 55)	AD (n = 54)	*p*
Age, years	78.0 (73.0–83.0)	78.0 (74.0–83.0)	82.0 (77.0–85.0)	0.008
Female, %	64	71	63	0.623
≤Primary education, %	60	53	77	0.023
**Cognitive and functional measures (partly inherent to group definitions) ^†^**				
S-MMSE	29.0 (27.0–29.0) [n = 53]	25.0 (24.0–27.0) [n = 54]	19.5 (16.8–25.0) [n = 40]	<0.001
Katz ADL	6.0 (5.0–6.0)	5.0 (5.0–6.0)	4.0 (3.0–5.0)	<0.001
Lawton IADL	8.0 (7.0–8.0)	7.0 (4.0–8.0)	1.0 (0.0–5.0)	<0.001
MNA-SF	12.0 (11.0–14.0)	12.0 (9.0–13.0)	9.0 (6.0–11.0)	<0.001
GDS-15	3.0 (1.0–6.0)	4.0 (2.0–7.0)	5.0 (3.0–6.0)	0.019
Three-word recall	2.0 (2.0–3.0)	2.0 (1.0–2.0)	0.0 (0.0–1.0)	<0.001
**Comorbidities, %**				
Diabetes mellitus	31	29	30	0.977
Hypertension	65	75	52	0.046
COPD	15	7	7	0.340
Osteoporosis	36	33	30	0.755
Hyperlipidaemia	16	16	15	0.968
Coronary artery disease	18	16	30	0.187
Heart failure ^‡^	7	4	2	0.361
Depression	5	7	6	0.905
Asthma	7	5	2	0.410
Stroke ^‡^	2	2	7	0.201
Polypharmacy (≥5 drugs), %	62	67	76	0.280
**Laboratory and indices, median (IQR)**				
Total protein (g/L)	70.0 (67.0–73.8)	70.0 (67.0–73.0)	67.0 (63.0–70.0)	<0.001
Albumin (g/L)	44.0 (42.0–45.0)	44.0 (41.0–45.0)	42.0 (39.0–44.0)	0.002
ALT (U/L)	19.0 (17.0–23.0)	16.0 (13.0–19.8)	17.0 (13.0–21.0)	0.003
eGFR (mL/min/1.73 m^2^)	64.5 (54.0–75.2)	64.0 (51.0–73.0)	71.0 (49.0–82.0)	0.710
Creatinine (mg/dL)	0.90 (0.80–1.10)	0.91 (0.80–1.19)	0.90 (0.70–1.08)	0.494
CRP (mg/L)	3.60 (2.30–5.60)	2.50 (1.40–3.90)	2.60 (1.30–4.55)	0.057
HbA1c (%)	5.8 (5.6–6.2)	5.8 (5.5–6.3)	5.6 (5.3–6.3)	0.121
Glucose (mg/dL)	95.0 (86.5–114.0)	92.5 (85.2–117.2)	92.0 (83.0–119.5)	0.704
Haemoglobin (g/dL)	13.0 (12.1–14.1)	12.6 (11.8–14.4)	12.9 (11.7–13.8)	0.666
Vitamin B12 (pg/mL)	401.5 (317.5–632.8)	364.0 (289.0–456.0)	373.0 (318.0–548.0)	0.245
Folate (ng/mL)	9.0 (7.0–12.0)	8.0 (6.0–11.0)	7.0 (6.0–11.0)	0.118
25-OH vitamin D (ng/mL)	29.0 (13.8–37.8)	25.5 (17.2–36.8)	27.0 (17.2–38.0)	0.855
Corrected calcium (mg/dL)	9.50 (9.30–9.80)	9.40 (9.10–9.60)	9.30 (9.00–9.60)	0.027
TSH (mIU/L)	1.90 (0.96–2.75)	1.67 (1.03–2.24)	1.41 (0.70–1.82)	0.073
PTH (pg/mL)	53.0 (35.0–66.5)	54.0 (41.0–73.0)	54.0 (35.0–77.5)	0.808
BMD T-score, L1–L4	−1.5 (−1.9–−0.3)	−0.9 (−1.6–−0.3)	−1.4 (−2.3–−0.2)	0.355
BMD T-score, femoral neck	−2.1 (−2.3–−1.6)	−1.7 (−2.3–−1.1)	−1.6 (−2.6–−1.0)	0.461

Values are reported as median (IQR) or %. ^‡^ S-MMSE was not administered in 17 participants due to the severity of cognitive impairment; group cell sizes appear in brackets. No floor value was assigned. *p* from Kruskal–Wallis (continuous) or chi-square (categorical); education compared as an ordinal level. Shaded *p* indicates nominal *p* < 0.05 (before FDR). ^†^ S-MMSE, Katz, Lawton, MNA-SF, and three-word recall may contribute to clinical group definition and should be read as group characterization. ^‡^ Advanced or decompensated heart failure was excluded; a stable history of heart failure was recorded as a comorbidity. Prior stroke was not an exclusion criterion unless the clinical and neuroimaging evaluation supported vascular dementia as the primary diagnosis. Comorbidity coding was verified by internal cross-checks. Polypharmacy was defined from the recorded medication-count category (≥5 medications). Erythrocyte sedimentation rate was excluded (high missingness).

**Table 2:** Serum ATG5 by cognitive group and primary models.

	Normal	MCI	AD
ATG5 ng/mL, plate 1, median (IQR)	12.5 (10.5–15.5)	11.1 (8.9–17.8)	10.5 (9.3–12.2)
ATG5 ng/mL, plate 2, median (IQR)	6.4 (5.5–8.5)	6.7 (5.6–8.8)	5.8 (4.8–8.0)
ATG5 within-plate z, median	−0.15	−0.17	−0.46
Panel B (supportive): pooled plate-adjusted Tobit regression (n = 162)		GMR (95% CI)	*p*
Panel A (primary): plate-stratified Tobit (adj. age, sex)		GMR (95% CI)	*p*
Plate 1 (n = 82), MCI vs. normal		0.96 (0.72–1.26)	0.758
Plate 1 (n = 82), AD vs. normal		0.81 (0.62–1.07)	0.143
Plate 1, group omnibus (Wald, 2 df)		χ^2^ = 2.45	0.293
plate 2 (n = 80), MCI vs. normal		1.19 (0.90–1.58)	0.217
plate 2 (n = 80), AD vs. normal		0.95 (0.71–1.28)	0.738
plate 2, group omnibus (Wald, 2 df)		χ^2^ = 2.66	0.264
MCI (vs. normal)		1.091 (0.896–1.327)	0.386
AD (vs. normal)		0.892 (0.730–1.091)	0.266
ELISA plate 2 (vs. plate 1)		0.530 (0.451–0.623)	<0.001
Age		0.994 (0.981–1.006)	0.300
Male sex		1.049 (0.883–1.246)	0.584
Group omnibus (Wald, 2 df)		χ^2^ = 3.896	0.143
Residual sigma = 0.5172 (geometric CV = 55%)		—	—
Plate × group interaction (LR, 2 df)		χ^2^ = 1.058	0.589

Kruskal–Wallis *p* = 0.095 for within-plate z-standardised ATG5. The plate-stratified Panel A constitutes the primary analysis, with Panel B serving a supportive role. n = 162; 6 right-censored observations. Reported values are geometric mean ratios, accompanied by 95% confidence intervals, after back-transformation from the log scale. No significant cognitive-group effect emerged in either analytical stratum, and no plate-by-group interaction was detected (*p* = 0.589).

**Table 3:** Age- and sex-adjusted partial Spearman correlations between serum ATG5 and clinical/laboratory variables.

Variable	Partial ρ	*p*	FDR *p*	n
S-MMSE	+0.283	<0.001	0.010	139
Lawton IADL	+0.197	0.014	0.052	155
Katz ADL	+0.121	0.131	0.184	156
MNA-SF	+0.140	0.083	0.144	154
GDS-15	−0.155	0.074	0.144	133
Three-word recall	+0.140	0.105	0.163	135
Folate	+0.255	0.002	0.015	142
Albumin	+0.196	0.015	0.052	155
Corrected calcium	+0.177	0.028	0.078	155
Haemoglobin	+0.150	0.063	0.144	155
eGFR	+0.010	0.905	0.905	150
CRP	−0.096	0.264	0.284	138
Vitamin B12	−0.107	0.194	0.247	150

ATG5 is within-plate z-standardised. FDR by Benjamini–Hochberg across the full panel of 13 variables. After correction, only S-MMSE and folate remained significant (FDR *p* = 0.010 and 0.015, respectively); Lawton IADL, albumin, and corrected calcium were nominal only.

**Table 4:** Sensitivity analyses for the serum ATG5–S-MMSE association.

Model/Stratum	ρ or β	95% CI	*p*
Within normal cognition	+0.469	(+0.216–+0.662)	<0.001
Within MCI	+0.361	(+0.095–+0.579)	0.009
Within clinically diagnosed AD	+0.021	(−0.297–+0.334)	0.900
Homogeneity across strata (Fisher-z, Q)	Q = 5.01	—	0.082
Pooled (age, sex)	+0.283	(+0.122–+0.429)	<0.001
Group-demeaned (within-group association)	+0.281	(+0.121–+0.428)	<0.001
+ education	+0.295	(+0.134–+0.441)	<0.001
+ education, albumin	+0.259	(+0.095–+0.409)	0.002
+ education, total protein	+0.281	(+0.112–+0.434)	0.001
+ education, albumin, folate	+0.292	(+0.121–+0.447)	0.001
+ education, albumin, folate, MNA-SF	+0.255	(+0.080–+0.414)	0.005
Linear regression (age, sex)	β = 0.0442	(0.0092–0.0791)	0.014
Cubic spline vs. linear (LR test)	χ^2^ = 2.933	—	0.402

ATG5 is within-plate z-standardised. The association persisted after adjustment for education and for folate and was of similar magnitude in the linear model (β = 0.044, *p* = 0.014). The pooled models were fitted using n = 139, whereas the most fully adjusted model included 121, according to covariate completeness; estimates for specific strata derived from 49 participants with normal cognition, 51 with MCI and 39 with clinically diagnosed AD.

**Table 5:** Secondary analyses: independent association and discrimination of within-plate standardised serum ATG5.

Analysis	ATG5 OR (95% CI)	*p*	ATG5-Alone AUC (95% CI)
Clinically diagnosed AD vs. normal cognition or MCI, logistic (adj. age, sex, and education)	0.75 (0.50–1.12)	0.157	0.60 (0.51–0.70)
MCI + AD vs. normal, logistic (adj. age, sex, and education)	0.97 (0.69–1.36)	0.854	0.56 (0.47–0.65)
Base model (age, sex, and education)—AD vs. others	—	—	AUC 0.700
Base + ATG5—AD vs. others	—	0.141 (LR)	AUC 0.712 (ΔAUC +0.012)
Base model (age, sex, and education)—MCI + AD vs. normal	—	—	AUC 0.601
Base + ATG5—MCI + AD vs. normal	—	0.854 (LR)	AUC 0.601 (ΔAUC +0.000)

OR, odds ratio; AUC, area under the receiver-operating-characteristic curve. To avoid circularity, cognitive and functional variables that feature in clinical classification were excluded from the logistic models, which instead utilised within-plate z-standardised ATG5. Events per variable ≈ 13–26; maximum variance inflation factor 1.4. Additional folate adjustment did not materially change the ATG5 estimate (clinically diagnosed AD vs. normal cognition or MCI OR 0.69, *p* = 0.124). Overall adjusted model AUCs were 0.71 and 0.60, driven mainly by age and education rather than ATG5. Because absolute ATG5 values could not be compared across the two plates, no diagnostic cut-off was established.

## Data Availability

The data presented in this study are available from the corresponding author upon reasonable request, subject to institutional and ethical restrictions.

## Supplementary Materials

The following supporting information can be downloaded at https://www.mdpi.com/article/10.3390/medicina62091722/s1. Table S1: Analytical characteristics of the ATG5 ELISA assay and plate-specific quality-control information.

## Abbreviations

ATG5	Autophagy-Related Protein 5
MCI	Mild Cognitive Impairment
AD	Alzheimer’s Disease
S-MMSE	Standardized Mini Mental State Examination
ADL	Activities of Daily Living Scale
IADL	Instrumental Activities of Daily Living Scale
NINCDS-ADRDA	National Institute of Neurological and Communicative Disorders and Stroke–Alzheimer’s Disease and Related Disorders Association
DSM-5	Diagnostic and Statistical Manual of Mental Disorders, Fifth Edition
MRI	Magnetic Resonance Imaging
ADL	Activities of Daily Living
GDS-15	15-item Geriatric Depression Scale
MNA-SF	Mini Nutritional Assessment Short Form
BMI	Body Mass Index
ALT	Alanine Aminotransferase
HbA1c	Haemoglobin A1c
CRP	C-Reactive Protein
