# Supplementary material for: Serum Autophagy-Related Protein 5 and Clinically Defined Cognitive Status in Older Adults: A Plate-Stratified Cross-Sectional Study Across: Normal Cognition, Mild Cognitive Impairment, and Alzheimer’s Disease
Source: Medicina (Kaunas). 2026 Sep 7;62(9):1722. doi: 10.3390/medicina62091722 (PMC13609047; doi:10.3390/medicina62091722)
Supplement: Supplementary file 1 [file medicina-62-01722-s001.zip › medicina-4459630-supplementary.pdf]

Supplementary Material

Serum Autophagy-Related Protein 5 and Clinically Defined Cognitive Status in Older Adults:A Plate-Stratified Cross-Sectional Study Across Normal Cognition, Mild Cognitive Impairment, and Alzheimer's Disease

Supplementary Table S1. Analytical characteristics of the ATG5 ELISA assay and plate-specific quality-control information.

Assay characteristics were obtained from the manufacturer's product manual (SunLong Biotech, catalogue SL2770Hu). Because serum was assayed at the manufacturer-recommended 1:5 dilution, the on-plate detection range of 0.1–10 ng/mL corresponds to a reportable serum range of 0.5–50 ng/mL; the calibrators actually run, however, spanned 0.75–9 ng/mL on plate, giving a calibrated serum range of 3.75–45 ng/mL. Six samples reading above the highest calibrator (45 ng/mL in serum) were right-censored in the primary analysis. The two columns correspond to the two 96-well plates, which were run from a single kit lot and calibrated against standards from that lot, in the same laboratory by the same operator under nominally identical conditions; the manufacturer's specifications listed above apply identically to both. Only the calibration curve and blank optical density recorded on the day, the replication of the calibrators (read in duplicate on plate 1 and in singlicate on plate 2, with all samples run in singlicate on both plates), and any laboratory-specific sample handling differ, and these values are given in the table above.

**Assay characteristic**	**Plate 1**	**Plate 2**
Analyte	Autophagy-related protein 5 (ATG5)	Autophagy-related protein 5 (ATG5)
Sample type	Serum	Serum
Manufacturer	SunLong Biotech Co., Ltd, Hangzhou, China	SunLong Biotech Co., Ltd, Hangzhou, China
Catalogue number	SL2770Hu (96 tests); Lot 20250704	SL2770Hu (96 tests); Lot 20250704
Assay method / format	Sandwich ELISA; HRP–TMB detection; OD at 450 nm	Sandwich ELISA; HRP–TMB detection; OD at 450 nm
Assay range (on plate)	0.1–10 ng/mL	0.1–10 ng/mL
Reportable range for serum (after 1:5 dilution)	0.5–50 (manufacturer); 3.75–45 (calibrators run)	0.5–50 (manufacturer); 3.75–45 (calibrators run)
Top standard / calibrators	13.5 ng/mL stock; curve 0.75–9 ng/mL	13.5 ng/mL stock; curve 0.75–9 ng/mL
Analytical sensitivity (LOD)	0.01 ng/mL	0.01 ng/mL
Intra-assay CV (manufacturer specification)	< 10%	< 10%
Inter-assay CV (manufacturer specification)	< 12%	< 12%
Sample dilution	1:5 (10 μL serum + 40 μL diluent)	1:5 (10 μL serum + 40 μL diluent)
Kit storage	2–8 °C; shelf life 6 months	2–8 °C; shelf life 12 months from manufacture
Samples above the highest calibrator (45 ng/mL), right-censored, n	5	1
QC-flagged samples (lipemic/clotted), n	2	4

Assay characteristic	Plate 1	Plate 2
Plates run from this kit lot	2 (plates 1 and 2, same kit lot)	2 (plates 1 and 2, same kit lot)
Samples per plate; replicate structure	84 samples, singlicate; calibrators and blank in duplicate	80 samples, singlicate; calibrators and blank in singlicate
Calibration curve model and r^2	Four-parameter logistic (log concentration, linear signal); $r^2 = 0.997$	Four-parameter logistic (log concentration, linear signal); $r^2 = 0.998$
Zero-standard (blank) optical density	0.078 and 0.073 (duplicate wells)	0.079 (single well)
Venepuncture to centrifugation, to freezing	Serum separated and frozen on the day of collection; exact intervals not recorded	Serum separated and frozen on the day of collection; exact intervals not recorded
Storage temperature and duration	−20 °C; assayed within 6 months of collection	−20 °C; assayed within 6 months of collection
Freeze–thaw cycles before assay	None; thawed once on the day of assay	None; thawed once on the day of assay
Operator blinded to cognitive group	Not documented; samples were allocated across plates interleaved with respect to diagnostic group	Not documented; samples were allocated across plates interleaved with respect to diagnostic group

Values are manufacturer specifications from the SL2770Hu product manual (sandwich ELISA; on-plate range 0.1–10 ng/mL, sensitivity 0.01 ng/mL, intra-assay CV < 10%, inter-assay CV < 12%; serum assayed at 1:5 dilution; the calibrators actually run were 9, 6, 3, 1.5 and 0.75 ng/mL on plate, giving a calibrated serum range of 3.75–45 ng/mL). In the analysis, six observations exceeded the highest calibrator (45 ng/mL) and were modeled as right-censored (five on plate 1, one on plate 2); quality-control–flagged samples (two on plate 1, four on plate 2) were examined in a sensitivity analysis. Both plates were run with reagents from a single kit lot, so the manufacturer's caution against mixing reagents from different lots does not apply; the two plates are analysed separately because of the observed between-plate scale difference (geometric mean ratio 0.53, 95% CI 0.45-0.62), roughly four times the manufacturer's stated inter-assay CV of below 12%. Both plates were run from a single kit lot (SunLong Biotech, Cat. SL2770Hu, Lot 20250704).

Note. This Supplementary file accompanies the main manuscript. Supplementary Table S1 is descriptive and hypothesis-free.
